# The INECO Frontal Screening tool differentiates behavioral variant - frontotemporal dementia (bv-FTD) from major depression

**DOI:** 10.1590/S1980-57642013DN70100006

**Published:** 2013

**Authors:** Natalia Fiorentino, Ezequiel Gleichgerrcht, María Roca, Marcelo Cetkovich, Facundo Manes, Teresa Torralva

**Affiliations:** 1 Institute of Cognitive Neurology (INECO), Buenos Aires, Argentina.; 2 Institute of Cognitive Neurology (INECO), Buenos Aires, Argentina. Institute of Neurosciences, Buenos Aires, Argentina.; 3 Institute of Cognitive Neurology (INECO), Buenos Aires, Argentina. Institute of Neurosciences, Buenos Aires, Argentina. Laboratory of Neuroscience, University of Diego Portales, Chile.

**Keywords:** frontotemporal dementia, major depression, executive dysfunction

## Abstract

Executive dysfunction may result from prefrontal circuitry involvement occurring in both neurodegenerative diseases and psychiatric disorders. Moreover, multiple neuropsychiatric conditions, may present with overlapping behavioral and cognitive symptoms, making differential diagnosis challenging, especially during earlier stages. In this sense, cognitive assessment may contribute to the differential diagnosis by providing an objective and quantifiable set of measures that has the potential to distinguish clinical conditions otherwise perceived in everyday clinical settings as quite similar.

**OBJECTIVE:**

The goal of this study was to investigate the utility of the INECO Frontal Screening (IFS) for differentiating bv-FTD patients from patients with Major Depression.

**METHODS:**

We studied 49 patients with bv-FTD diagnosis and 30 patients diagnosed with unipolar depression compared to a control group of 26 healthy controls using the INECO Frontal Screening (IFS), the Mini Mental State Examination (MMSE) and the Addenbrooke's Cognitive Examination-Revised (ACE-R).

**RESULTS:**

Patient groups differed significantly on the motor inhibitory control (U=437.0, p<0.01), verbal working memory (U=298.0, p<0.001), spatial working memory (U=300.5, p<0.001), proverbs (U=341.5, p<0.001) and verbal inhibitory control (U=316.0, p<0.001) subtests, with bv-FTD patients scoring significantly lower than patients with depression.

**CONCLUSION:**

Our results suggest the IFS can be considered a useful tool for detecting executive dysfunction in both depression and bv-FTD patients and, perhaps more importantly, that it has the potential to help differentiate these two conditions.

## Abstract (pt)

A disfunção executiva pode resultar de envolvimento do circuito pré-frontal que ocorre em doenças neurodegenerativas e distúrbios psiquiátricos. Além disso, várias condições neuropsiquiátricas, podem apresentar sobreposição de sintomas comportamentais e cognitivos, tornando o diagnóstico diferencial um desafio, especialmente durante as fases iniciais. Neste sentido, a avaliação cognitiva pode contribuir para o diagnóstico diferencial, fornecendo um conjunto de medidas objetivas e quantificáveis com potencial para distinguir as condições clínicas percebidas em ambientes clínicos comuns como bastante similar.

**OBJETIVO:**

O objetivo deste estudo foi o de investigar a utilidade do Rastreio Frontal INECO (IFS) em diferenciar pacientes bv-FTD de pacientes com depressão maior.

**MÉTODOS:**

Foram estudados 49 pacientes com diagnóstico de bv-FTD e 30 pacientes com diagnóstico de depressão unipolar, que foram comparados com um grupo controle de 26 controles saudáveis usando o IFS, o Mini Exame do Estado Mental (MMSE) e Exame Cognitivo de Addenbrooke-revisado (ACE-I).

**RESULTADOS:**

Os grupos de pacientes diferiram significativamente no controle inibitório motor (U=437,0, p<0,01), memória de trabalho verbal (U=298,0, p<0,001), a memória de trabalho espacial (U=300,5, p<0,001), provérbios (U=341,5, p<0,001) e no controle inibitório verbal (U=316,0, p<0,001), com pacientes com bv-FTD tendo pontuação significativamente menor do que os pacientes com depressão.

**CONCLUSÃO:**

Nossos resultados sugerem que o IFS pode ser considerado uma ferramenta útil para detectar a disfunção executiva em depressão e pacientes bv-FTD e, talvez mais importante, que tem o potencial de ajudar na diferenciação dessas duas condições.

## INTRODUCTION

Frontotemporal dementia (FTD) is a progressive neurodegenerative disease, which primarily affects the frontal and anterior temporal lobes and is associated with heterogeneous underlying pathologies.^1,2^ Several major clinical syndromes have been identified, including behavioral variant (bv-FTD), temporal variant (tv-FTD), and motor variant (mv-FTD) FTDs.^3^ Patients who develop the bv-FTD have early and prominent impairments in executive functions^4^ and profound changes in decision-making^5,6^ and in several aspects of moral^6^ and social cognition.^7^ However, general cognitive functions such as memory, language and praxis may be relatively spared,^8,9^ especially during the earlier stages of the disease. Some patients can present deficits in complex functions such as planning, judgment, reasoning, problem-solving, organization, attention, abstraction, and mental flexibility.^10^ Patients can also predominantly present emotional blunting, namely apathy, inertia and loss of volition.^11,12^ As these behavioral symptoms progress, flattened and poorly-regulated affection can be readily observed along with decreasing interest in usual social, recreational, occupational and creative pursuits.^13^

During the early stages, conventional brain imaging techniques (CT, MRI and SPECT) can be rather insensitive;^14-17^ making diagnosis extremely challenging. In addition, initial symptoms can be subtle and perplexing, as these patients do not show striking cognitive deficits, but rather symptoms that can be interpreted as adjustment problems, stress, or lapses of judgment and self- control.^13^ This also explains why bv-FTD patients are usually misdiagnosed with psychiatric disorders, such as late-onset bipolar disorder, schizophrenia, or depression among others.^11-18^

The difficulty differentiating bv-FTD from non-demented psychiatric patients lies in the fact that executive functions are the most frequently impaired in these disorders. For instance, patients with major depression may present with bv-FTD, like cognitive and behavioral^11^ symptoms. For this reason, cognitive assessment can contribute to depicting a clearer diagnostic picture, as specific cognitive processes may help distinguish between different diseases. In fact, cognitive screening tools such as the INECO Frontal Screening (IFS)^19^ have been designed to specifically assess executive functions, especially when classical tests such as the Trail Making Test or the Wisconsin Card Sorting Test used exclusively may fail to detect the dysexecutive syndrome shown by these patients in everyday life or when their administration is limited due to the lack of human, time, or physical resources. Although the IFS was originally designed to determine frontal dysfunction in patients with dementia, its ability to discriminate bv-FTD from psychiatric patients has not yet been tested.

In the present study, we investigated the utility of the IFS for differentiating bv-FTD patients from patients with major depression, as they constitute a clinical population majorly affected by executive dysfunction.^11^ We hypothesized that performance on the IFS by both clinical groups would be impaired relative to controls, but that bv-FTD patients would obtain even lower scores than patients with depression.

## METHOD

**Participants.** A total of 105 participants were included in this study, 49 of whom were patients diagnosed with bv-FTD, 30 diagnosed with unipolar depression, and 26 healthy controls. All subjects were recruited from a larger pool of participants at the Institute of Cognitive Neurology (INECO). Healthy controls were examined with a comprehensive neuropsychological and neuropsychiatry evaluation, and had no history of either neurological or psychiatric disorders, including traumatic brain injury or substance abuse. Patients underwent a standard examination battery including neurological, neuropsychiatric and neuropsychological examinations and a MRI-SPECT and were followed over time to assess progression of the disease. They were classified into their corresponding clinical groups by either fulfilling new consortium criteria for probable bv-FTD^20^ or DSM-IV criteria for depression.^21^ Patients fulfilling both criteria were excluded from the study in order to avoid potential additive effects of two underlying yet concomitant disorders.

**Procedure.** The study was previously approved by the ethics committee of the Institute of Cognitive Neurology (INECO) following international regulations established for human research subjects. All participants were evaluated with an extensive neuropsychological battery. Data for this study were obtained from the following tests:

- Mini Mental State Examination (MMSE).^22^ This is the globally most popular and widely-used brief cognitive status screening tool for bedside assessment.
- Addenbrooke's Cognitive Examination-Revised (ACE-R).^23^ The ACE-R was developed to incorporate the items of the MMSE but further assess other cognitive domains, and has shown superior sensitivity and specificity in the detection of cognitive impairment, especially in the earlier stages of dementia.
- INECO Frontal Screening (IFS).^19^ As explained before, the IFS was designed to assess different aspects of executive functioning, thus assessing the domains neglected by the MMSE and ACE-R. It includes the following tasks:

1. Motor programming (3 points).^24,25^ This subtest requires the patient to perform the Luria series, "fist, edge, palm" by initially copying the administrator, and subsequently doing the series on his/her own and then repeating the series 6 times alone. Depending on the extension of frontal lesion or degeneration, some patients may not be able to complete the series in the correct order on their own, and others may not even be able to copy it. If subjects achieved 6 consecutive series by themselves, the score was 3, if they achieved at least 3 consecutive series on their own, the score was 2; if they failed to achieve at least 3 consecutive series alone, but achieved 3 when copying the examiner, the score was 1; otherwise score was 0.
2. Conflicting instructions (3 points).^25^ Interference.^25^ Subjects were asked to tap the table once when the administrator tapped it twice, or to tap the table twice when the administrator tapped it only once. To ensure the subject had clearly understood the task, a practice trial was performed in which the administrator first hit the table once, three times in succession, and then twice, three more times. After the practice trial, the examiner completed the following series: 1-1-2-1-2-2-2-1-1-2. If subjects committed no errors, the score was 3; if they committed one or two errors, the score was 2; while for more than two errors, the score was 1, unless the subject copied the examiner at least 4 consecutive times, in which case the score was 0. Patients with frontal lesions tend to imitate the examiner's movements, ignoring the verbal instruction.
3. Go - No go (3 points).^25^ This task was administered immediately after test 2. Subjects were told that now, when the test administrator tapped the table once, they should tap it once as well, but when the examiner taps twice, they should do nothing. To ensure the subject had clearly understood the task, a practice trial was performed in which the administrator tapped the table once, three times in succession, and then twice, three more times. After the practice trial the examiner completed the following series: 1-1-2-1-2-2-2-1-1-2. If subjects committed no errors, the score was 3; for one or two errors the score was 2; for more than two errors the score was 1, unless the subject copied the examiner at least 4 consecutive times, in which case the score was 0.32
4. Backwards Digit Span (6 points).^26^ For this task, subjects were asked to repeat a progressively lengthening string of digits in the reverse order. Two trials were given at each successive list length, beginning at 2 and continuing to a maximum of 7. If subjects passed either trial at a given list length, then the next length was administered. The score was the number of lengths at which the subject passed either trial, with a maximum of 6.
5. Verbal working memory (2 points).^26^ The patient was asked to list the months of the year backwards, starting with December. If subjects committed no errors, the score was 2; for one error, the score was 1; otherwise the score was 0. This task evaluates the same function as the previous subtest but with a slightly different load since the series is highly overlearned for most individuals.
6. Spatial Working Memory (4 points).^27^ In this task, the examiner presented the subject with 4 cubes and pointed at them in a given sequence. The subject was asked to repeat the sequence in reverse order. There were 4 trials, with sequences of two, three, four and five cubes respectively. Score was number of correctly completed sequences.
7. Abstraction capacity (Proverb interpretation) (3 points).^26^ Patients with frontal lesions exhibit difficulties on abstract reasoning tasks. Reasoning is most frequently clinically assessed in one of two ways, namely, with either similarities or proverb interpretation tasks. The latter was chosen for this screening test, since patients with frontal lesions usually have difficulties in stepping back from the concrete facts to find their abstract meaning. In this task 3 proverbs were read to the subjects who were asked to explain their meaning. For each proverb a score of 1 was given when the subject gave an adequate explanation, and a score of 0.5 for a correct example. Otherwise the score was 0. The three proverbs were chosen specifically for this demographic population based on their high frequency in oral speech
8. Verbal inhibitory control (6 points).^28^ This task, based on the Hayling test, measures a subject's capacity to inhibit an expected response. Materials were 6 sentences, each missing the last word and constructed to strongly constrain what the word should be. In the first part (3 sentences), subjects were read each sentence and asked to complete it correctly, as quickly as possible. In the second part (remaining 3 sentences), subjects were asked for a completion that was syntactically correct but unrelated to the sentence in meaning. Only the second part was scored. For each sentence, a score of 2 was given for a word unrelated to the sentence, a score of 1 for a word semantically related to the expected completion, and a score of 0 for the expected word itself. Example: "An eye for an eye, a tooth for a ...(table)..." By presenting an identical structure during both phases, this subtest is potentially capable of efficiently evaluating two executive function components (initiation and inhibition) in relation to a unique symbolic verbal form.^29^

The IFS has a maximum possible total score of 30 points and takes less than 10 minutes to administer and score. A 25-point cutoff score has shown a sensitivity of 96.2% and a specificity of 91.5% in detecting patients with dysexecutive syndrome (bv-FTD).^19^

**Statistical analysis.** Demographic and clinical information, as well as neuropsychological test performances, were compared between the groups using one-way ANOVAs with Bonferroni post hoc analyses when appropriate. When data was not normally distributed, U Mann-Whitney tests were used to compare two groups at a time. When analyzing categorical variables (e.g. gender), the Freeman-Halton extension of the Fisher exact probability test for 2x3 contingency tables was used. The ability of the MMSE, ACE-R and IFS to discriminate healthy controls from patients diagnosed with either bv-FTD or depression was determined using a receiver operating characteristic (ROC) curve analysis. All statistical analyses were performed using the SPSS 17.0 software package.

## RESULTS

Demographic profile and total scores on tests of general cognitive status are summarized in Table 1. A significant difference was found for age (F_2,102_=10.1, p<0.001), with depression patients being slightly younger than both controls (p=0.001) and bv-FTD patients (p<0.01). Nonetheless, neither years of formal education (F_2,102_=0.96, p=0.39) nor gender (χ^2^=4.7, p=0.10) differed significantly between the groups. Both the MMSE (F_2,102_=33.1, p<0.001) and the ACE-R (F_2,102_=27.1, p<0.001) differed across the groups. In both cases, however, bv-FTD scored significantly lower than both groups (p<0.001 for MMSE and ACE-R), but the performance of controls and patients with depression did not differ significantly (p=0.36 for MMSE and p=0.06 for ACE-R). These differences remained after covarying for age (MMSE: F_2,98_=25.2, p<0.001; ACE-R: F_2,98_=31.6, p<0.001)

**Table 1:** Demographic profile and performance on cognitive screening tools. Values are expressed as Mean (SD).

	bv FTD(n=49)	Depression (n=30)	Control (n=26)
Age	69.69 (8.70)	60.17 (11.53)	69.23 (8.94)
Years of education	13.24 (4.67)	14.17 (3.89)	14.46 (2.23)
Gender (F : M)	24 : 25	22 : 8	14 : 12
CDR 1	(0.78)	NA	NA
BDI-II	12 (11.09)	26 (12.7)	NA
MMSE	23.76 (4.96)	28.11 (1.80)	29.65 (0.48)
ACE-R	68.22 (19.11)	85.67 (10.88)	95.54 (3.04)
IFS	13.43 (7.26)	21.10 (5.12)	27.48 (1.61)

MMSE: Mini Mental State Examination; ACE-R: ACE-R: Addenbrooke's Cognitive Examination- Revised; IFS: INECO Frontal Screening; CDR: Clinical Dementia Rating Scale; BDI-II: Beck Depression Inventory II. NA: not available.

**Figure 1:**
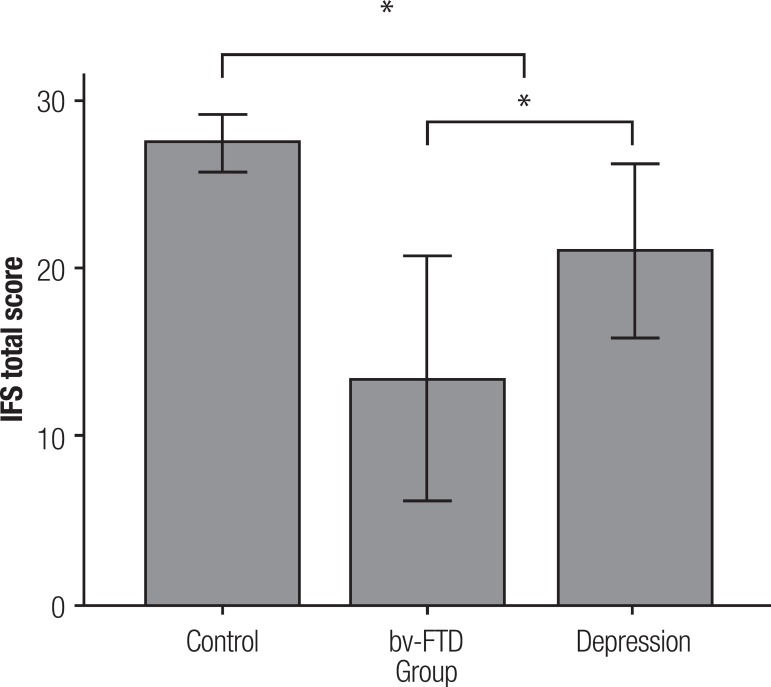
Comparison of IFS total score performance across groups. Error bars represent SD. *p<0.05.

**Figure 2:**
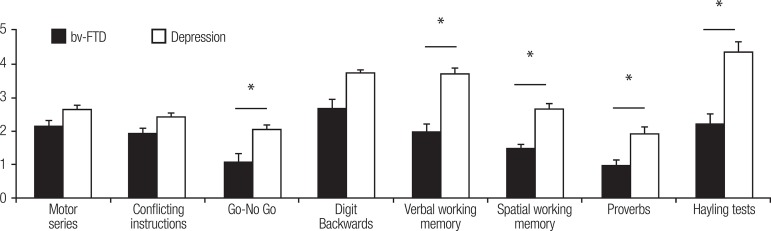
Performance comparison of bv-FTD vs. depression patients on IFS subtests. Error bars represent SEM. * p<0.05.

Total score on the IFS differed significantly between the groups (F_2,102_=53.4, p<0.001) even after covarying for age (F_2,98_=56.0, p<0.001), and as shown by Figure 1, not only did controls score significantly higher than bv-FTD (p<0.001) and depression (p<0.001) patients, but the two clinical groups also differed in their performance by almost 8 points on average (p<0.001). Further analyses revealed that controls exhibited significantly higher scores than both clinical groups on each sub-test of the IFS (all p<0.01). In turn, patient groups differed significantly on the motor inhibitory control (U=437.0, p<0.01), verbal working memory (U=298.0, p<0.001), spatial working memory (U=300.5, p<0.001), proverbs (U=341.5, p<0.001) and verbal inhibitory control (U=316.0, p<0.001) subtests, with bv-FTD patients scoring significantly lower than patients with depression (Figure 2). No significant differences were found between clinical groups on the motor series (U=615.5, p=0.17), conflicting instructions (U=624.5, p=0.22), and digit backward span (U=569.0, p=0.13) subtests.

**Figure 3:**
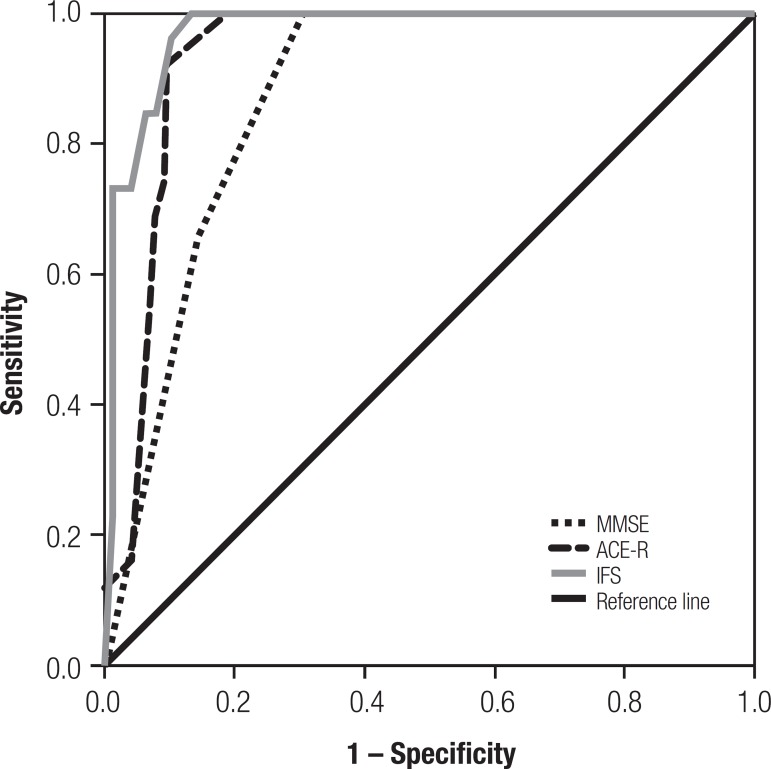
ROC curve for controls vs. patient groups (bv-FTD and depression). The superior discriminatory accuracy of the IFS over the MMSE and ACE-R is revealed by its larger area under the curve.

Analysis of the area under the curve generated by ROC curves revealed that the IFS had superior discriminatory accuracy (AuC=0.97, SE=0.01) than the MMSE (AuC=0.88, SE=0.03) and the ACE-R (AuC=0.93, SE=0.03) in discriminating healthy controls from patient groups (Figure 3). The same superior discriminatory accuracy of the IFS was confirmed in its ability to distinguish bv-FTD from patients with depression specifically (IFS: AuC=0.84, SE=0.04; MMSE: AuC=0.78, SE=0.05; ACE-R: AuC=0.79, SE=0.05)

## DISCUSSION

Executive dysfunction may result from prefrontal circuitry involvement occurring both in neurodegenerative diseases^4,10^ and psychiatric disorders. Moreover, multiple neuropsychiatric conditions may present with overlapping behavioral characteristics, making differential diagnosis challenging, especially during the earlier stages. In this scenario, cognitive assessment may contribute to the differential diagnosis by providing an objective and quantifiable set of measures that has the potential to distinguish clinical conditions otherwise perceived in everyday clinical settings as quite similar.

In the particular case of bv-FTD, patients are known to present executive dysfunction and behavioral deficits, which include disinhibition, impulsivity, loss of insight and apathy.^7,30^ Although executive dysfunction is common in bv-FTD, executive dysfunction can also be present in other psychiatric conditions that can mimic bv-FTD such as major depression.^31-33^ Even if neuropsychological assessment could potentially contribute to distinguishing these disorders, a complete neuropsychological assessment is not always readily available to physicians. In this regard, various easy-to-administer screening tools have been designed to provide brief instruments that can help to detect cognitive deficits: while some screening tools have been created to detect general cognitive deficits, such as the MMSE and the ACE-R, others have been designed to specifically assess executive functions, such as the IFS. The aim of the present study was to test whether the IFS, as an executive screening tool, had superior ability than the MMSE and the ACE-R in the differentiation of patients with bv-FTD from patients with major depression.

Our results showed that performance of both bv-FTD and major depression patients on the IFS was significantly lower than that of controls. Our results also showed that the IFS led to superior discriminatory accuracy for distinguishing bv-FTD from major depression than both general screening tools (MMSE and ACE-R). These results support the use of the IFS not only in patients with frontal degenerative pathologies (bv-FTD), but also for patients with psychiatric disorders such as depression. Further studies recruiting larger populations of psychiatric patients with diagnoses that mimic bv-FTD will be crucial to determine whether the superior discriminatory accuracy of the IFS is actually statistically significant.

Our results are consistent with previous literature showing executive dysfunction both in bv-FTD^4,7,19,34,35^ and depression.^31-33^ Furthermore, the fact that bv-FTD showed lower performance than the depressive group on inhibitory control, working memory tests, and proverb interpretation seems consistent with the early and prominent impairments in executive function described in bv-FTD reflecting the early structural involvement of frontal lobe structures.^7,35,19^ These findings are in strong agreement with previous findings using the IFS to compare the performance of patients with Alzheimer's disease (AD) and bv-FTD using the IFS where the same subtests (with the exception of visual working memory) differed significantly between these patient groups.^18^ The relatively superior performance of depressive patients on these tasks may also reflect the functional and potentially reversible involvement of frontal circuitry.

Although previous studies have examined the efficacy of other screening tests for detecting executive dysfunction in frontal lobe pathologies such as the Frontal Assessment Battery (FAB),^25^ this is the first study to show that an executive screening tool is able to differentiate between psychiatric and neurologic conditions, such as depression and bv-FTD. Our results indicate that the IFS is an adequate screening tool for detecting executive impairments both in neurologic and psychiatric conditions, suggesting its potential utility in a wide variety of neuropsychiatric populations.

Overall, this study showed that the IFS can be considered a useful tool for detecting executive dysfunction in both depression and bv-FTD patients and, perhaps more importantly, that it has the potential to help differentiate these two conditions; while very low scores might be indicative of bv-FTD, medium-low scores might indicate major depression. These findings are crucial for clinical settings where comprehensive neuropsychological assessment is difficult and the need for a differential diagnosis between depression and bv-FTD can be decisive. This is especially true considering that the potential treatment and care management is radically different in the two diseases and that the instrument used in this study has shown solid psychometric properties.^19,36^

Although a large number of studies have demonstrated the relevance of early detection of specific impairments for social and emotional aspects of cognition in frontal lobe dysfunction conditions, our study indicates that the utilization of a brief and straightforward tool sensitive to frontal dysfunction can also be very helpful, especially when limited time is available.
